# Validating the Physical Activity and Leisure Motivation Scale (PALMS)

**DOI:** 10.1186/1471-2458-14-909

**Published:** 2014-09-03

**Authors:** Keyvan Molanorouzi, Selina Khoo, Tony Morris

**Affiliations:** Sports Centre, University of Malaya, Kuala Lumpur, Malaysia; Institute of Sport, Exercise and Active Living (ISEAL), College of Sport and Exercise Science, Victoria University, Melbourne, Australia

**Keywords:** Motives for participating in physical activity, Intrinsic motivation, Extrinsic motivation, Physical activity, PALMS, Reliability, Validity

## Abstract

**Background:**

Although there is abundant evidence to recommend a physically active lifestyle, adult physical activity (PA) levels have declined over the past two decades. In order to understand why this happens, numerous studies have been conducted to uncover the reasons for people’s participation in PA. Often, the measures used were not broad enough to reflect all the reasons for participation in PA. The Physical Activity and Leisure Motivation Scale (PALMS) was created to be a comprehensive tool measuring motives for participating in PA. This 40-item scale related to participation in sport and PA is designed for adolescents and adults. Five items constitute each of the eight sub-scales (mastery, enjoyment, psychological condition, physical condition, appearance, other’s expectations, affiliation, competition/ego) reflecting motives for participation in PA that can be categorized as features of intrinsic and extrinsic motivation based on self-determination theory. The aim of the current study was to validate the PALMS in the cultural context of Malaysia, including to assess how well the PALMS captures the same information as the Recreational Exercise Motivation Measure (REMM).

**Method:**

To do so, 502 Malaysian volunteer participants, aged 18 to 67 years (mean ± SD; 31.55 ± 11.87 years), from a variety of PA categories, including individual sports, team sports, martial arts and exercise, completed the study.

**Results:**

The hypothesized 8-factor model demonstrated a good fit with the data (CMIN/DF = 2.820, NFI = 0.90, CFI = 0.91, RMSEA = 0.06). Cronbach’s alpha coefficient (α = 0.79) indicated good internal consistency for the overall measure. Internal consistency for the PALMS subscales was sound, ranging from 0.78 to 0.82. The correlations between each PALMS sub-scale and the corresponding sub-scale on the validated REMM (the 73-item questionnaire from which the PALMS was developed) were also high and varied from 0.79 to 0.95. Also, test-retest reliability for the questionnaire sub-scales was between 0.78 and 0.94 over a 4-week period.

**Conclusions:**

In this sample, the PALMS demonstrated acceptable factor structure, internal consistency, test-retest reliability, and criterion validity. It was applicable to diverse physical activity contexts.

## Background

The link between regular physical activity (PA) and physical and psychological health has been well documented in the literature [1–3]. The most important benefits of regular PA include reduced prevalence of many diseases as well as decreased mortality [4–7]. Individuals of all ages can gain an array of physical, psychological, social, and emotional benefits from PA [8–10]. Despite the established benefits of regular PA, a large proportion of the population in the United States [11], Europe [12] and Malaysia [13] do not participate in adequate PA to gain these health benefits and are still not sufficiently active or maintain a sedentary lifestyle.

For these reasons, researchers, health professionals, and policy makers have all sought to explore why some people are physically active, whereas others are not. Although the antecedents to participation in PA are highly complex [14], the most crucial reason for people to be physically active during their spare time is motivation. Motivation not only affects PA participation, but is also a critical factor in exercise adherence [15–18].

PA is defined as any movements carried out by the skeletal muscles that require energy above the basal metabolic rate [19]. Exercise is a sub-category of PA that incorporates planned, structured, and repetitive movements. Sport is another sub-category of PA which includes structured competitive situations that are governed by rules [20]. Most researchers have focused on examining motivation in competitive sport [21, 22] or adopted measures of motivation developed for competitive sport [17, 23]. Others have examined exercise and developed measures of motivation for that context [24, 25]. There is a need to validate measures of motivation that can be applied to non-competitive PA, including organized exercise and informal leisure activities, as well as competitive sport, so that researchers examining reasons for participation in PA can study the full range of activities with the same measure, thus, facilitating comparison. Here we refer to PA except when discussing research that focused on sport or exercise.

Researchers have adopted various approaches to develop standardized instruments to investigate and study participation motives [26]. Two approaches have typified the development of most measures of motives for participation in PA. Theoretical approaches involve the development of a questionnaire structure and the generation of items on the basis of a theory of motivation. Atheoretical approaches are based on studies in which researchers identify reasons for participation in PA by asking participants why they participate, develop items based on participants’ responses, and determine underlying factors statistically. The following instruments measure motives for PA based on a particular theory: the Sport Motivation Scale (SMS; [23]), the Exercise Motivation Scale (EMS; [24]), the Exercise Motivation Inventory (EMI; [25]), the Motivation for Physical Activity Measure (MPAM; [27]), the Motivation for Physical Activity Measure – Revised (MPAM-R; [28]) and the Perception of Success Questionnaire for Exercise (POSQ-E; [29]). The developers of these instruments have based their content on different theoretical approaches to the notion of motivation for PA, resulting in a variety of motives in the different instruments. For example, the SMS, EMS and EMI are built on Self-Determination Theory (SDT), particularly the intrinsic and extrinsic components of SDT [30]. Ryan and colleagues [27, 28] developed the MPAM and its expanded version, the MPAM-R, specifically to examine Deci and Ryan’s SDT. The MPAM measures interest/enjoyment and competence motives that reflect intrinsic motivation and health is considered an extrinsic motive. In the revision of this scale to produce the MPAM-R, the health motive was divided into fitness and appearance motives, and a social motive was added, so there are three extrinsic motives. The POSQ-E, however, is heavily based on goal orientation or task/ego motivation theory [31, 32]. As a result, most of these measures of motivation include incentives for exercise or sport that are pertinent only to the specific theory that underpins their development. Hence, they do not cover the motives and incentives that participants suggest when they are asked for the reasons they participate in physical activity in an open format, such as an unstructured interview. To put it differently, an open interview makes it possible for individuals to explain, provide instances and demonstrate personal motives for participation. What is more, a number of theoretically-based questionnaires were discovered to serve weak psychometric features. For instance, Markland and Ingledew [33] proposed that the EMI not be applied for the evaluation of the degrees of intrinsic motivation because of problems which are conceptually or operationally-oriented (it was understood that 6 alternatives were seen to be unneeded or superfluous, thus the incremental R for every alternative over 4 for every provided aspect was small). The EMI did not affirm motives that are relevant to competitive dimensions. Likewise, EMI did not evaluate a number of clear fitness-oriented causes for exercising, such as endurance and strength. Besides, the subscales pertinent to wellbeing and health concentrated on ill-health which ignores motives that are not only health-oriented, but also positively based [33]. Even as a 5-factor expansion of the MPAM, the MPAM-R [28] suggested a restricted number of motives. The POSQ was created specifically for the measurement of just two goal orientations: one is the task or mastery orientation and the other is the ego or competition orientation. Interestingly, the obtained differences on the POSQ explained approximately 50% of the data variance, showing that further variables should be taken into consideration [28]. Moreover, the meticulous aim of design of the POSQ was to be used in the domain of competitive sports. As a result, the first approach, based on theories, in particular SDT or goal orientation theory, has generated questionnaires with a relatively narrow focus, thus, lacking comprehensiveness. Research suggests that movement towards health could be a more positive, intrinsically-oriented motivational force [34, 35].

A principal example of the atheoretical approach is the work of Gill, Gross, and Huddleston [36]. They adopted an empirical method to develop a measure of motives for participation in youth sports. They asked youth sport participants to state their reasons for participation, based on open-ended questions. Using the acquired information, Gill et al. [36] devised a 30-item questionnaire, named the Participation Motivation Questionnaire (PMQ). The PMQ was later administered to 1,138 youth participants at a multi-sport summer camp. After conducting an exploratory factor analysis (EFA), Gill et al. distinguished eight factors to be used in the PMQ, namely achievement, team (affiliation/social), fitness, energy release, to be with others, skill, friends, and fun [36]. A number of researchers in the sport and exercise realm have used versions of the PMQ to examine the motives for participation in a range of contexts, such as youth specific sport [37–39], youth multi-sport [40, 41] and multi-sport across the lifespan [22]. Although the numerous versions of the PMQ do cover a breadth of motives for participation in PA, a stable version of the questionnaire has yet to be established with a set number of items that can be used in various PA contexts [17]. The biggest drawback in the PMQ is that it is descriptive and is not associated with an established theory of motivation.

To address the limitations of previous instruments as well as the drawbacks of both the theory-based and the atheoretical approaches, Rogers et al. [26] created a new instrument based on both empirical and theoretical approaches. Initially, a qualitative study was performed in which 11 in-depth, semi-structured interviews were conducted with exercise participants aged 21 to 50 years. To focus on achievement goals, they used the terms “success” and “goals” throughout the interview, and avoided the terms “motive”, “motivation”, or “reasons” for participation. This distinction was made because although goals and motives are often used interchangeably, they are conceptually distinct. More specifically, a goal is a specific external target, whereas a motive is an internal drive influencing behavior [42]. Through inductive content analysis, they identified 13 first-order themes that were further reduced to 7 meaningful concepts (competition/ego, extrinsic rewards, social health, physical health, psychological health, mastery, and enjoyment), which were then grouped under the general dimensions of intrinsic and extrinsic motivation. Based on the data from the qualitative study and compared to the results of the 50-item version of the PMQ [22], the MPAM [27], and the MPAM-R [28], a 73-item measure was developed with responses on 5-point Likert scales. This was called the Recreational Exercise Motivation Measure (REMM) [43]. Rogers examined the reliability and validity of the 73-item REMM with a sample of 750 recreational exercisers. First-order factor analysis revealed eight factors, namely mastery, enjoyment, psychological condition, physical condition, appearance, others’ expectations, affiliation, and competition/ego. Cronbach’s alpha coefficients of the eight sub-scales were 0.77 to 0.92, showing sound internal consistency, and test-retest reliabilities were 0.58 to 0.84 [43]. Because the eight factors were not orthogonal, that is, they were correlated, a second-order factor analysis was performed on participants’ factor scores for the eight first-order factors. This produced three fundamental factors consistent with the intrinsic-extrinsic motivation components of SDT, namely an intrinsic motivation factor, comprising mastery and enjoyment, an extrinsic body-mind motives factor, comprising psychological condition, physical condition, and appearance, and an extrinsic social motives factor, including others’ expectations, affiliation, and competition/ego. Rogers then conducted a comparison study with 250 recreational sports participants and found that the REMM produced very similar reliability and validity statistics, indicating that the measure is acceptable for use with participants in competitive sports and non-competitive physical activities.

The REMM has been applied successfully in research. Aaltonen et al. [18] showed that several motives on the REMM distinguished between pairs of twins one of whom had been active for 30 years and the other inactive for that period. In each case, REMM scores were significantly higher for the active twins than their inactive siblings. Caglar, Ermin, and Demir [44] reported that females rated health as a more important motive than males and young adults rated health, appearance, social/affiliation, and skill motives more highly than adolescents. In spite of the fact that the REMM has shown promise as a measure of motives for participation in sport and PA, it has limitations. Further refinement of the original 73-item version of the REMM would be of value [45]. A concern with the REMM was that administration of a scale of this length might not always be convenient in sport and PA contexts. In fact, impatience or boredom might affect the answers given by respondents. To address these shortcomings, a short-form version of the REMM was created. Rogers and Morris [45] proposed that it would be fruitful to develop a shorter version of the REMM that was easier to administer and quicker to complete than the original.

A short-form version of the REMM was developed based on a combination of empirical and theoretical factors. First, Morris and Rogers [46] determined the structure and length that is appropriate for the short form version of the REMM. Second, they conducted item analysis, including the examination of means and standard deviations, skewness and kurtosis, factor loadings, item-sub-scale correlations, and item-deleted alpha coefficient values. They utilized this information to guide the selection of items for the short-form measure. Finally, the five strongest items were selected on all eight factors in the REMM to create a 40-item measure, the Physical Activity and Leisure Motivation Scale (PALMS), which we expected would be intrinsically equivalent to the REMM.

Given that the PALMS was derived from the REMM, it is plausible to accept that the PALMS, like the REMM, would have sound psychometric features. In order to ensure that the PALMS is a reliable and valid instrument, it should be tested on a large, international sample from a range of activities. Chowdhury [47] administered the PALMS to 202 volunteer sport, exercise, and martial arts participants, aged 18 to 71 years, from various organizations, clubs, and leisure centers in Australia. Results of a confirmatory factor analysis (CFA) indicated that the PALMS had a robust factor structure (CMIN/DF = 2.22; NFI = 0.95; CFI = 0.97; RMSEA = 0.078). Zach, Bar-Eli, Morris and Moore [48] translated the PALMS into Hebrew (PALMS-H) and validated it with 678 recreational exercise participants, aged 9 to 89 years, who exercised regularly in Israel. They reported that the PALMS-H demonstrated good internal consistency for each of the sub-scales, ranging from 0.63 to 0.96.

Building on the foundation laid by the Australian and Israeli studies, the objective of the present study was to examine the psychometric properties of PALMS as a measure of leisure-time PA in a diverse sample of exercisers and sport participants within the population of Malaysia. More specifically, in this study we examined the internal consistency, test-retest reliability and factor structure of the PALMS. The purpose of correlating the PALMS and the REMM was to assess how well the PALMS captures the same information as the REMM. Investigating these properties in a diverse Asian population like Malaysia not only allowed us to examine whether the PALMS is appropriate for use in research and practice in various Asian cultures, but also gave us the opportunity to test the robustness of the PALMS for use within a wide range of cultures.

## Methods

### Ethics statement

The study was approved by the Institute of Research Management and Monitoring, University of Malaya and the Sports Centre Research Committee. Participation in the study was voluntary and all adult participants provided written consent to participate in the study.

### Participants

In this study, a sample of 502 volunteers (259 males, 243 females) aged 18 to 67 years (mean ± SD; 31.55 ± 11.87 years) who participated in regular PA (at least 150 minutes of moderate- to vigorous-intensity PA per week) in the last six months participated in this study. Participants reported that their main PA included swimming, tennis, soccer, cycling, basketball, taekwondo and tai chi. All participants resided in Malaysia. They comprehended spoken and written English.

### Measures

#### Demographics form

Participants reported key demographic variables, including gender, age, and race. They also reported their primary PA, and the level, frequency, duration and intensity of activity and extent of their participation per week. The items in regular PA were structured to provide separate domain-specific scores for walking, moderate intensity and vigorous intensity activity within each of the leisure time PA. To calculate regular PA, only the activities lasting at least 150 minutes of moderate- to vigorous-intensity PA per week were taken into account. We are looking at PA in a later study.

In the present study participants were instructed to respond to the PALMS and the REMM with reference to their main physical activity. To provide information about what this activity was, we asked participants to state their main PA in the Demographics Form. We cite that information in describing the demographics of the sample. No further analysis in this study addresses type of physical activity. In a related study we have examined the relationship between motives and type of physical activity.

#### Recreational Exercise Motivation Measure (REMM)

The 73-item REMM measures eight motives for participation in recreational exercise, namely mastery, enjoyment, psychological condition, physical condition, appearance, other’s expectations, affiliation, competition/ego, on a 5-point Likert scale ranging from 1 (strongly disagree) to 5 (strongly agree). The range for each sub-scale of the REMM varies because the number of items varies from 7 to 13. In each case, the range is represented by the lowest score of 1 multiplied by the number of items on that sub-scale to the highest score of 5 multiplied by the number of items on the sub-scale. Thus, for the 7-item sub-scale the range is 7 to 35, whereas for the 13-item sub-scale the range is 13 to 65 [45].

#### Physical Activity and Leisure Motivation Scale (PALMS)

The 40-item PALMS (Table 1) assesses the same eight motives for participation in PA as the REMM. It was developed as a short form of the REMM by selecting the five items with the strongest psychometrics on each of the eight sub-scales. Responses to the PALMS are made on the same 5-point Likert scales as used with the REMM. The range of each PALMS sub-scale is 5 to 25 because each sub-scale has five items [46].

**Table 1:** **Items and sub-scales in the PALMS**

Item no.	Item	Sub-scale
6	Because I perform better than others	Competition/Ego
17	To be best in the group	Competition/Ego
27	To work harder than others	Competition/Ego
29	To compete with others around me	Competition/Ego
39	To be fitter than others	Competition/Ego
11	To define muscle, look better	Appearance
23	To improve body shape	Appearance
32	To improve appearance	Appearance
36	To lose weight, look better	Appearance
40	To maintain trim, toned body	Appearance
1	To earn a living	Others expectations
7	Because I get paid to do it	Others expectations
18	To manage medical condition	Others expectations
21	Because people tell me I need to	Others expectations
26	Because it was prescribed by doctor, physio	Others expectations
4	Because I enjoy spending time with others	Affiliation
8	To do activity with others	Affiliation
20	To do something in common with friends	Affiliation
30	To talk with friends exercising	Affiliation
38	To be with friends	Affiliation
10	Because it helps maintain a healthy body	Physical condition
12	Be physically fit	Physical condition
15	To maintain physical health	Physical condition
28	Because it keeps me healthy	Physical condition
33	To improve cardiovascular fitness	Physical condition
2	Because it helps me relax	Psychological condition
9	To better cope with stress	Psychological condition
14	To get away from pressures	Psychological condition
22	Because it acts as a stress release	Psychological condition
35	To take mind off other things	Psychological condition
5	To get better at an activity	Mastery
16	To improve existing skills	Mastery
19	To do my personal best	Mastery
24	To obtain new skills/activities	Mastery
31	To keep current skill level	Mastery
3	Because it’s interesting	Enjoyment
13	Because it makes me happy	Enjoyment
25	Because it’s fun	Enjoyment
34	Because I enjoy exercising	Enjoyment
37	Because I have a good time	Enjoyment

#### Shortened Marlowe-Crowne Social Desirability Scale (MCSDS)

The shortened MCSDS is a 13-item short form of the original MCSDS [49]. The MCSDS was developed to assess individuals’ need to respond in a socially desirable way. The shortened MCSDS has been shown to be psychometrically sound, but much quicker and easier to complete than the original scale [49, 50]. To examine whether people respond to a questionnaire, in this case the PALMS, to look good, scores on the questionnaire are correlated with scores on the MCSDS. A moderate to high positive correlation with the MCSDS would indicate socially desirable responding on the PALMS.

### Procedure

Participants were recruited from various recreational parks and facilities from May to July 2012. Their participation was voluntary. Information sheets were distributed for all participants. If they agreed to participate after reading the information sheet, completion of the questionnaires was considered to indicate consent. Thus, this was a convenience sample in which participants were accessed through local recreation facilities. One implication of this is that there was no systematic control over the gender, age, and regular physical activity patterns of the participants. Participants completed all the measures in English. Malaysia is a country in which the national language, Malay, is widely spoken, while several other languages associated with the large ethnic Indian and Chinese populations are also spoken by substantial numbers of people. Nonetheless, because of its British colonial heritage, English language education starts in primary school in Malaysia and a substantial proportion of the population from all ethnic backgrounds speak English well, even if it is not their “native” language. For this study, the PALMS and other measures were administered in English. To ensure that participants’ responses were based on sound understanding of the instructions, the items, and the response format, participants were screened for their capacity to read and comprehend English at a high level. We examined the questionnaire responses made by participants and did not identify indications in those responses that suggested lack of comprehension for the participants included in the analyses cited in this paper. Based on standard questionnaire checking processes, any participants whose responses showed signs of such response patterns were eliminated from the sample before the analyses were conducted. The number eliminated was small.

In order to eliminate order effects, half the participants were given the demographic form and the PALMS followed by the MCSDS. After a short break, the participants were given the REMM. The other half completed the demographic form and the REMM followed by a break after which the MCSDS and PALMS were completed. Social desirability scales are all different, so there is no “index score”. The developers of each scale indicate the cut-off point or range of scores that reflect social desirability responding. There are few social desirability scales. The MCSDS is the most widely used measure. One way to use the MCSDS in practice or research is to exclude participants who score above the cut-off point. Another conventional way to apply the MCSDS is to use the scores from a large sample to check whether people respond to other questionnaires in socially desirable ways. This is done by correlating the MCSDS with the target scale. A strong correlation indicates a systematic pattern of responding to the target questionnaire that is consistent with the responses given to the MCSDS. For example, given that high scores on the MCSDS reflect that participants are responding in a socially desirable way, a positive correlation with high scores on the PALMS would indicate that participants are systematically responding in a socially desirable way on the PALMS. Conversely a low correlation between the MCSDS and the PALMS would indicate that participants are not responding systematically to the PALMS in a socially desirable way [50]. The 502 respondents took 20–25 minutes to complete the demographic form and the three questionnaires. Test-retest reliability of the PALMS was examined by administering the PALMS twice, four weeks apart, with a sub-sample of 92 of the participants who volunteered for the main study. The sub-sample comprised 49 males and 43 females aged 18–55 years (mean ± SD; 36.65 ± 9.94 years).

### Statistical analysis

Reliability was assessed by means of test-retest reliability to examine stability over time, and alpha coefficients to examine internal consistency. In terms of criterion validity, each of the eight sub-scales of the PALMS was correlated using Spearman’s rank-order correlation coefficient with the corresponding sub-scale on the REMM. Pearson’s product–moment correlations between the sub-scales of the PALMS and the MCSDS were also examined to determine whether participants were responding to the PALMS in socially desirable ways. Effect sizes are used as indicators of practically meaningful differences. With reference to the correlations cited in this paper, it is not conventional in psychological research to cite confidence intervals. Instead, psychologists consider effect sizes as indicators of practically meaningful differences. Cohen [51] indicated that Pearson’s Product Moment Correlation Coefficient (r) is itself a measure of effect size. Values of r can be interpreted in the same way as Cohen’s d, a common indicator of effect size. Thus, values of .2-.3 are considered to be small effect sizes, those around .5 are medium, and values of .7-.8 are viewed as large effect sizes. We have now interpreted the measures of association in terms of effect size.

**Figure 1:**
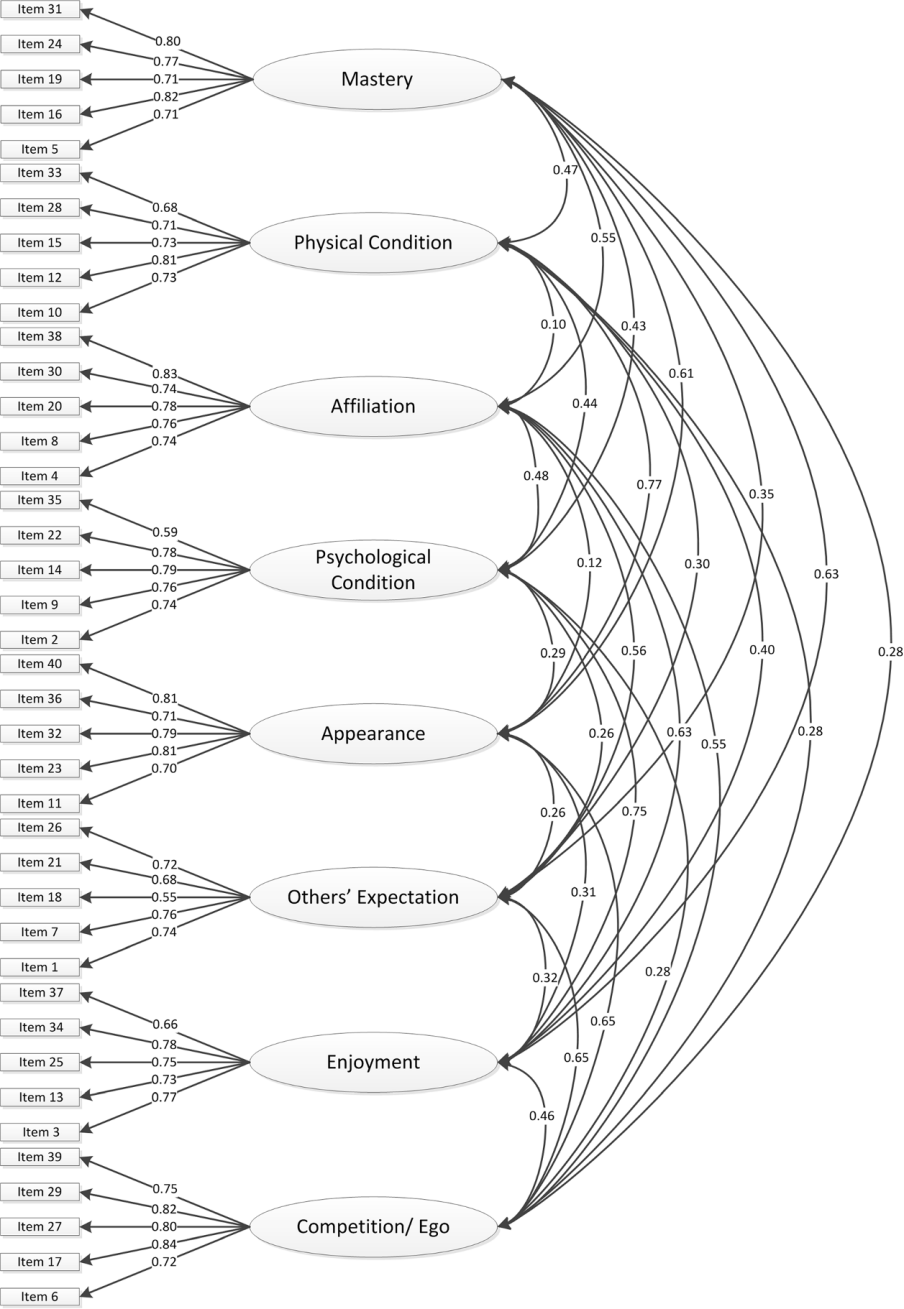
**Measurement model for PALMS.**

There are few social desirability scales. The MCSDS is the most widely used measure. One way to use the MCSDS in practice or research is to exclude participants who score above the cut-off point. Another way to use the MCSDS is to use the scores from a large sample to check whether people respond to other questionnaires in socially desirable ways. This is the way in which the MCSDS is used in the present study. Thus, the MCSDS was correlated with the sub-scales of the PALMS. A strong correlation indicates a systematic pattern of responding to the PALMS that is consistent with the responses given to the MCSDS. For example, given that high scores on the MCSDS reflect that participants are responding in a socially desirable way, a positive correlation with high scores on the PALMS would indicate that participants are systematically responding in a socially desirable way on the PALMS, that is, they are reporting high motivation sub-scale scores to look good. Conversely a low correlation between the MCSDS and the PALMS would indicate that participants are not responding systematically to the PALMS in a socially desirable way [50].Prior to performing CFA, preliminary analyses were conducted on the univariate distributions of all the variables to verify whether they were normally distributed with low degrees of skewness and kurtosis. CFA was then conducted through AMOS 20.0 on the eight sub-scales of the PALMS. Each sub-scale included in the path diagram in the CFA was measured by its own set of observed indicators. Maximum likelihood was the method of estimation used for the models. In the present study, a path diagram was drawn to depict the relationship between the sub-scales (8 factors) and the observed variables (items on the PALMS), as shown in Figure 1 in the Results section. In this path diagram, we proposed an 8-factor model, based on the results of the first-order exploratory factor analysis of the REMM. The factors are the eight PALMS sub-scales mastery, enjoyment, psychological condition, physical condition, appearance, others’ expectations, affiliation, and competition/ego. The analysis examined the paths from the five items designated to measure each motive sub-scale to that motive sub-scale or factor. The assumptions of normality were also checked.

To evaluate the fit of the models, we considered four indices of model fit: the minimum discrepancy divided by the degrees of freedom (CMIN/DF ratio), two comparative fit indexes, the comparative fit index (CFI) and the Tucker-Lewis index (TLI), and the root mean square error of approximation (RMSEA). Values of CMIN/DF less than 5 are considered reasonable for macro constructs [52]. The CFI and TLI reflect the degree to which the sample variances and covariances are reproduced by the hypothesized model structure. CFI and TLI values above 0.90 usually indicate acceptable fit. RMSEA was used to assess approximate fit, preferably with values less than or equal to 0.06 [53].

## Results

### Internal consistency, criterion validity and reliability of the PALMS

The PALMS demonstrated good internal consistency with a Cronbach’s alpha (α) of 0.82. The internal consistency values of the eight PALMS sub-scales are presented in Table 2. They were generally high, the lowest being 0.78 for Mastery and Competition/ego, demonstrating that all the sub-scales had strong internal consistency in this sample. Also, Spearman’s rho indicated a strong positive correlation between the REMM and the PALMS (*r*_*s*_ = 0.86, *p* < .001). Furthermore, the Spearman’s rho correlations between each PALMS sub-scale and the corresponding REMM sub-scale showed high correlations (r_s_ = 0.79 to 0.95), providing evidence that the PALMS is intrinsically equivalent to the REMM, which lends support to the criterion validity of the eight PALMS sub-scales (see Table 2). The eight sub-scales of the PALMS also showed high test-retest correlations, ranging from r_s_ = 0.78 to 0.94, supporting the stability of the components of the measure over time. Thus, effect sizes for the correlations between the REMM and the PALMS all reflected large effects. Table 2 also shows the Pearson’s product–moment correlation coefficients between each of the sub-scales of the PALMS and the MCSDS. These correlations are all close to zero, reflecting very small effect sizes, indicating that the PALMS did not encourage socially desirable responses in this study.

**Table 2:** **Internal consistency, test-retest correlation, criterion validity and Pearson’s product–moment correlations of the PALMS**

Sub-scales	PALMS	PALMS	PALMS &REMM	PALMS & MCSDS
	Internal consistency (α)	Test-retest correlation	Correlations (r _s_)	Pearson’s Product–moment correlations (r)
**Mastery**	0.78^*^	0.91^**^	0.83^*^	-0.09
**Physical condition**	0.82^*^	0.82^**^	0.89^*^	0.01
**Affiliation**	0.80^*^	0.91^**^	0.95^*^	0.05
**Psychological condition**	0.81^*^	0.88^**^	0.80^*^	-0.01
**Appearance**	0.81^*^	0.91^**^	0.86^*^	-0.05
**Others’ expectations**	0.82^*^	0.94^**^	0.88^*^	-0.04
**Enjoyment**	0.79^*^	0.83^**^	0.79^*^	0.04
**Competition/ego**	0.78^*^	0.78^*^	0.86^*^	-0.02

**Legend:** α = Cronbach’s alpha. *r*_*s*_ = Spearman’s rho.

**Correlation is significant at the 0.01 level (2-tailed).

*Correlation is significant at the 0.05 level (2-tailed).

### Confirmatory factor analysis

CFA was performed on responses to the PALMS questionnaire assessing the fit of the model depicted in Figure 1, in which each of the 40 items is shown with a path connecting it to the appropriate motive among the eight sub-scales (mastery, enjoyment, psychological condition, physical condition, appearance, other’s expectations, affiliation, competition/ego).

The CFA, which is presented in Table 3, yielded a good fit of this model to the data (CMIN/DF = 2.82, NFI = 0.90, CFI = 0.91, RMSEA = .06).

**Table 3:** **Model fit indices for the data collected using PALMS**

	N	CMIN	DF	CMIN/DF	NFI	CFI	RMSEA	(90% CI)
**Model**_**H**_	502	2007.758	712	2.820	0.899	0.909	0.060	0.057^_^0.063

**Legend:** Model _H_ = the hypothesized model. N = sample size. CMIN = minimum discrepancy. DF = degrees of freedom. NFI = normed fit index. CFI = comparative fit index. RMSEA = root mean square error of approximation. (90% CI) = lower boundary of a two-sided 90% confidence interval for the population and upper boundary of a two-sided 90% confidence interval for the population.

## Discussion

The results showed acceptable internal consistency reliability for the PALMS. Cronbach’s alpha values were comparable to those reported by other researchers, particularly Zach et al. [48] and Chowdhury [47]. Cronbach’s alpha values for all sub-scales of the PALMS were high. Based on statistical indexes, this means that the items consistently measure the factors with which they are associated. The PALMS sub-scales maintained high internal consistency reliability values despite being shorter than the corresponding sub-scales in the REMM. This is consistent with previous research [26, 47, 48]. These findings lend further support to the consistency of the items in the PALMS as representative of the sub-scales to which they have been attributed. This supports the internal consistency of the instrument for assessing participation motivation for PA. In addition, the test-retest reliability, measured by Spearman’s rank-order correlation coefficient, was high for all sub-scales, with the lowest value for competition/ego, reflecting a strong association between scores from administrations four weeks apart. The other seven PALMS sub-scales reflected very high associations over this substantial 4-week test-retest period. As test-retest reliability has not been examined in previous studies on this questionnaire, there are no test-retest values for the purpose of comparison. Thus, this is the first demonstration that the motives for participation measured by the PALMS are stable over a fairly long period of 4 weeks. The criterion validity of the PALMS was supported by Spearman’s rho, which indicated a strong positive correlation between the REMM and the PALMS overall, as well as high correlations between the corresponding sub-scales of the REMM and the PALMS. This provides evidence that the PALMS is intrinsically equivalent to the REMM, indicating strong support for the criterion validity of the PALMS as a measure of participation motivation that can be used to examine participation motives people nominate for their involvement in diverse PA contexts.

The very low correlations of each of the eight sub-scales of the PALMS with the MCSDS, which mostly approached zero, indicate that the PALMS did not encourage socially-desirable responses in this sample of participants within the largely recreational contexts in which they completed the questionnaires. This indicates that participants did not feel the need to respond to items in ways that they thought would make them look good. Evidence that the PALMS encourages honest responding is promising for the future use of the measure in diagnostic work related to motivation for PA.

It is noteworthy that the internal consistency, test-retest reliability over a 4-week period, and criterion validity of the PALMS in relation to the REMM were all sound in the present study given that the participants were all Malaysian residents completing the questionnaires in English. For most of these participants English was not their first language. This suggests, not only that the PALMS is robust, but that it is also clear and comprehensible enough to produce results that so closely mirror those found in the Australian sample, for whom English was their first language. The robustness found in the current study was also evident in the degree of consistency between the results found in the Hebrew translation of the PALMS in the study conducted in Israel and the English language version used in the present study and the Australian study.

In addition, results from the CFA on the 40 PALMS items revealed a desirable goodness-of-fit between the proposed 3-factor model and the data collected from this substantial sample of participants in diverse types of PA in the context of a large city in Malaysia. This is consistent with the eight sub-scale structure of the PALMS and also provides support for the construct validity of the PALMS, as reflected in previous research [47, 48]. Furthermore, the high, unmediated effects of the latent variables on the observed variables indicated that the items are actually measuring what they have been assigned to measure. Hence, the results reported here suggest that the hypothesized model in the current study fitted the data well, lending support to the initial validity of the PALMS. It can be claimed that the present results support the applicability of this questionnaire as a measure of a wide range of motives for participation in diverse PA contexts. The eight factors measured by the PALMS can be categorized as aspects of intrinsic motivation (mastery, enjoyment sub-scales) and extrinsic motives (the other six sub-scales). This is based both on the results of second-order factor analysis [45] and on SDT [54]. Further, the six extrinsic motives can be classified into two second-order factors, body-mind motives (psychological condition, physical condition, and appearance) and social motives (others’ expectations, affiliation, and competition-ego) [45]. In addition, each of the eight motivational sub-scales has implications for intentions and behavior related to PA. For example, a high score on the appearance sub-scale might reflect an intention to seek out PA that with improve body shape, such as weight training to build muscle or yoga to increase suppleness and flexibility. Similarly, a high score on affiliation could lead individuals to join football teams or weekend cycling clubs.

The PALMS demonstrated not only proper factor structure, initial validity, and reliability, but also showed that it is applicable to PA contexts. The obtained factor structure provided support for the SDT framework of motivational categories. Furthermore, the PALMS offers a more comprehensive analysis of participant motives than previous questionnaires that were based on either achievement goals or SDT, such as the MPAM-R. The factor structure within the PALMS could provide valuable information for health authorities and fitness professionals about the range of motives that people have for participation. This information can be applied to enhance exercise participation to fulfill a variety of purposes, not just health-based motives, which have traditionally been seen as important reasons for doing PA.

Like other studies, this one has a number of limitations and assets. Firstly, we gathered the data by self-report. However, previous studies on motivation for PA have generally used self-report and the results have shown acceptable reliability and validity. A second limitation is that the sizeable commitment of time to complete the demographic form and the three questionnaires could have caused fatigue or boredom, but high correlations between the PALMS and the REMM suggest that it is unlikely that responding was distorted by these factors. One further point to be considered is that the data in the present study was checked for missing values, so only responses from completed questionnaires were selected for analysis. The sample comprised a diverse range of people in terms of age, gender, and type of PA, but they were all from one country, Malaysia. Nonetheless, they do represent a culture that is quite different from the Australian culture in which the motivation questionnaires (REMM, PALMS) were developed. The REMM and PALMS have now been examined in Australia [45, 47], Turkey [44], Finland [18], Israel [48], and now Malaysia with a high degree of consistency and stability, suggesting that the underlying factors measured by these instruments are motives that apply across cultures and languages. This provides support for the factorial invariance of the PALMS. Caution in interpreting these results should reflect limitations in the design and measures. One limitation is that the data are cross-sectional and do not permit inferences about causality. In addition, all of the indices are based on self-report and subject to the potential for reporter bias. Participants were recruited by direct invitation to people exercising in public environments. They were given an information statement and if they agreed to participate completion of the questionnaires was considered to indicate consent. Thus, this was a convenience, sample in which participants were accessed through local recreation facilities. One implication of this is that there was no systematic control over the gender, age, and regular physical activity patterns of the participants. Also, acknowledging our statement in the Procedure section, it is recognized that the use of English language versions of the PALMS, REMM, and MCSDS questionnaires in the present study could be considered a limitation. We are confident from our screening of responses by the participants for all the questionnaires and the findings for all analyses, which are consistent with predictions, that participants included in the final sample clearly understood the content of the questionnaires and responded in a meaningful manner to the items in those measures.

In the present study the PALMS was shown to have good stability across four weeks. It is important for researchers to examine the long-term stability of the PALMS, so that it can be used to monitor changes in motives resulting from intentional interventions, which might last for several months, with confidence that changes observed do not reflect artifacts of the measuring instrument. Also, although previous work with the REMM in Finland and Turkey, as well as PALMS studies in Australia, Israel, and now Malaysia show promise for the universal nature of the motives measured by the PALMS, the PALMS should be further investigated in other contexts (e.g., different countries, languages, and/or activities and participants from other cultures). In addition, it should be noted that studies have been conducted recently in Malaysia to examine other aspects of the psychometric validity of the PALMS. In one study with a very large sample, discriminant function analyses indicated limited difference between males and females, more noteworthy differences between ages from adolescents to older adults, and important differences between types of physical activity (team ball sports, racquet sports, individual body-movement sports, exercise activities, martial arts). In another study with a large sample, PALMS motives are correlated with actual PA levels. Both studies provide further support for the construct validity of the PALMS. These studies will be published separately in the near future.

## Conclusion

The PALMS can be used as an instrument to help understand people’s motives for PA as the basis for recommending types of activity to which those individuals should be suited. In addition, the PALMS would then be suitable for research and applied work conducted around the world.

## Authors’ information

KM: PhD student at the Sport Centre, University of Malaya, Kuala Lumpur, Malaysia; lecturer at the Sport Science and Physical Education Faculty, Islamic Azad University Islamshahr Branch, Iran.

SK: Senior lecturer at the Sports Centre, University of Malaya, Kuala Lumpur, Malaysia

TM; Professor at the Institute of Sport, Exercise and Active Living (ISEAL), College of Sport and Exercise Science, Victoria University, Melbourne, Australia.

## Abbreviations

PA: Physical Activity
PALMS: Physical Activity and Leisure Motivation Scale
REMM: Recreational Exercise Motivation Measure
MCSDS: Shortened Marlowe-Crowne Social Desirability Scale
SDT: Self-determination Theory
CFA: Confirmatory Factor Analysis
RMSEA: Root Mean Square Error of Approximation
CFI: Comparative Fit Index
TLI: Tucker-Lewis Index.
